# Lockdown Amid COVID-19 Ascendancy over Ambient Particulate Matter Pollution Anomaly

**DOI:** 10.3390/ijerph192013540

**Published:** 2022-10-19

**Authors:** Muhammad Azher Hassan, Tariq Mehmood, Ehtisham Lodhi, Muhammad Bilal, Afzal Ahmed Dar, Junjie Liu

**Affiliations:** 1 Tianjin Key Lab of Indoor Air Environmental Quality Control, School of Environmental Science and Engineering, Tianjin University, Tianjin 300072, China; 2 College of Ecology and Environment, Hainan University, Haikou 570228, China; 3 Department of Environmental Engineering, Helmholtz Centre for Environmental Research—UFZ, D-04318 Leipzig, Germany; 4 The SKL for Management and Control of Complex Systems, Institute of Automation, Chinese Academy of Sciences, Beijing 100190, China; 5 School of Surveying and Land Information Engineering, Henan Polytechnic University, Jiaozuo 454000, China; 6 School of Environmental Science and Engineering, Shaanxi University of Science and Technology, Xi’an 710000, China

**Keywords:** COVID-19, health effects, lockdown, particulate matter (PM), transmission dynamics

## Abstract

Air is a diverse mixture of gaseous and suspended solid particles. Several new substances are being added to the air daily, polluting it and causing human health effects. Particulate matter (PM) is the primary health concern among these air toxins. The World Health Organization (WHO) addressed the fact that particulate pollution affects human health more severely than other air pollutants. The spread of air pollution and viruses, two of our millennium’s most serious concerns, have been linked closely. Coronavirus disease 2019 (COVID-19) can spread through the air, and PM could act as a host to spread the virus beyond those in close contact. Studies on COVID-19 cover diverse environmental segments and become complicated with time. As PM pollution is related to everyday life, an essential awareness regarding PM-impacted COVID-19 among the masses is required, which can help researchers understand the various features of ambient particulate pollution, particularly in the era of COVID-19. Given this, the present work provides an overview of the recent developments in COVID-19 research linked to ambient particulate studies. This review summarizes the effect of the lockdown on the characteristics of ambient particulate matter pollution, the transmission mechanism of COVID-19, and the combined health repercussions of PM pollution. In addition to a comprehensive evaluation of the implementation of the lockdown, its rationales—based on topographic and socioeconomic dynamics—are also discussed in detail. The current review is expected to encourage and motivate academics to concentrate on improving air quality management and COVID-19 control.

## 1. Introduction

Coronavirus disease 2019, known as COVID-19, is caused by severe acute respiratory syndrome coronavirus 2 (SARS-CoV-2). Although coronavirus epidemics in Wuhan, China, were detected in December 2019, it was officially confirmed as an outbreak on 11 February 2020 [1]. COVID-19 and particulate matter (PM) have a deep connection. PM is the sum of solid or liquid phase substances that are suspended in the air. PM is ubiquitous and comprised of chemicals (minerals, dust, polycyclic aromatic hydrocarbons (PAHs), organic matter, etc.) [2,3] and biological species (pollen, fungi, and bacteria) [4,5]. PM influences health, climate, cloud formation, ecology, and visibility through physicochemical reactions [6,7].

PM has various size fractions, from sub-nanometer clusters to millimeter-sized dust particulates. Particulates are generally divided into three groups based on their diameters, i.e., coarse, fine, and ultrafine PM [8,9]. PM_10_ (coarse PM with a 50% cut-off aerodynamic diameter of 10 µm), PM_2.5_ (fine PM with a 50% cut-off aerodynamic diameter of 2.5 µm), and PM_1_ (ultrafine PM with a 50% cut-off aerodynamic diameter of 1 µm). On the other hand, coronaviruses with single-stranded RNA are of a minute diameter, from 65–125 nm as a nucleic material, and vary in length from 26 to 32 kbs. Since tiny viral particles in the aerosol are suspended, particles such as avian influenza viruses, airborne in large amounts following dust storms in Asia, can be transported long distances from the origin of outbreaks [10,11]. Frontera et al. [12] addressed the fact that a highly polluted environment with such climatic conditions, distributed laterally (i.e., Asia), may promote longer stability of infectious particles in the air. In addition to direct individual dissemination, this would facilitate the indirect dissemination of SARS-CoV-2. Martelletti et al. [13] found the highest PM_10_ and PM_2.5_ levels among the northern Italian regions more impacted by COVID-19. These authors proposed that the PM could be a carrier of SARS-CoV-2. SARS has been found to spread along three common transmission routes: (i) 21% by long-distance aerosol, (ii) 29% through close contact among people via droplets, and (iii) 50% through surface contact [14]. Moreover, Setti et al. [15] provided evidence of SARS-CoV-2 RNA loaded on PM samples, suggesting a potential indicator of a resurgence of the pandemic via PM. Maintaining a social distance of 2 m may not be sufficient to protect individuals from COVID-19 infection, especially indoors and in polluted regions [16,17].

During a cycle of high smog, a metagenomic study in Beijing, China, evaluated the composition of air pollutant species. Multiple pathogens, including viruses, have been identified as sequences (0.1% in both PM_10_ and PM_2.5_). The number of respiratory pathogens increases with a rise in pollutant concentration. At a continental site with moderated pollution, the concentration of these particles varied with attitude: least in the stratosphere (<10 particles/cm^3^ at 20 km altitude) [18] and most in the troposphere (>1000 particles/cm^3^). Moreover, some urban areas showed over 1×10^5^ particles cm^−3^ [19]. Hence, the concentration of the virus could be higher around the breathing zone near ground level. Similarly, persons living in cities with elevated air pollution concentrations are more exposed to respirational disorders [20] and sensitive to pathogenic infections [21].

The relation concerning serious respiratory viral diseases as a cause of infection is well identified with air pollution in 10 to 20% of the global population [22]. Moreover, the viruses can live longer and become more active by attaching to PM, affecting an exacerbated immune system [23]. Therefore, an area with an elevated concentration of PM (PM_2.5_ and PM_10_) is assumed to be riskier for COVID-19 spreading.

Air pollutants such as microplastics, PM_2.5_ and PM_10_ can irritate the respiratory tract [21,24] and can worsen respiratory virus infections. The results in Italy [25] and in the United States (US) show that constant air pollutant exposure impedes recovery and contributes to severe and lethal conditions [26,27]. In that sense, Coccia et al. explored the mechanisms of COVID-19 spreading and prevention in the ecosystem to establish a potential strategy for coping with future coronavirus-like epidemics [28]. Inhaled environmental pollution impairs the safety of upper airways in the first line, primarily the cilia [29]. Moreover, Conticini et al. [30] studied whether populations in highly polluted areas such as Lombardia and Emilia Romagna are more vulnerable to death from COVID-19 because of their poorer initial health condition, triggered by air pollution. It was detected that higher air pollution concentration in Northern Italy must be considered as an additional factor of the high lethality in this region. Similarly, the association between PM_2.5_ and PM_10_ concentrations in other pollutants and COVID-19 cases identified in 120 cities in China was investigated by Zhu et al. [31]. In reported victims, significant relation between COVID-19 and air pollution has been recognized.

In Italy, which has one of the world’s highest death rates, the case study indicated that two processes activate the accelerated dynamics of spreading COVID-19 in specific environments: PM air pollution and high population density. The two key results are (1) the accelerated spreading dynamics of COVID-19 in Northern Italy is strongly connected to cities’ pollution, and (2) the cities with air pollution of more than 100 days, in terms of PM_10_, exhibited a higher average number of infected people (3340 cases). However, there are still many unanswered concerns regarding the link between PM pollution and COVID-19. For instance, in contrast to the aforementioned relation, Bontempi [32] studied PM_10_ concentration from 10 February to 27 March 2020 in Lombardy (Italy), several days before the sanitary emergency explosion. No direct connections between high PM_10_ levels and the dissemination of COVID-19 were found when data on concentrations in Lombardy and Piedmont were analyzed. To conclude, the COVID-19 pandemic may have paradoxically reduced overall deaths due to the enormous reduction in air contamination following quarantine and significantly reduced the deaths caused by air pollution itself [33].

Therefore, the current review covers the PM pollution dynamics in the COVID-19 era. We reviewed different studies which address PM pollution and COVID-19 cases, the impact of PM on COVID-19 cases, and the ascendancy of lockdowns on PM pollution anomaly in different cities of the world. We discussed the significant components of PM_10_ and PM_2.5_, tried to evaluate these components, and critically reviewed the studies which showed any positive or negative impact of COVID-19. In addition, since the lockdown situation in many major affected areas significantly reduced PM and its associated species, both negative and positive effects of the COVID-19 era on PM pollution are discussed. For a clear picture, the current review emphasizes the studies of countries greatly impacted by COVID-19 (China, USA, Italy, and India). Studies from other countries are also presented for comparison.

In addition, various meteorological factors and their impact on PM and COVID-19 are discussed in detail. Finally, this review has also demonstrated the health effects of PM and COVID-19 and related mechanisms. Therefore, we believe the present study will advance our understanding of PM pollution and how it interlinks with other health effects, such as COVID-19, which may be effective in efficient control and prevention strategies. Furthermore, the current study is also relevant to scholars and decision-makers examining the connections between infectious diseases around the world and PM pollution.

## 2. Impact of Lockdown on PM Mass Concentration

### 2.1. Inference of Lockdown on Emission Sources

The lockdown-based reduction of PM pollution showed complex phenomena, and many studies showed contradictory results. The lockdown decreased human activity by up to 90%, plus environmental emissions in Spain, the US, Italy, and Wuhan by nearly 30% [34]. Reducing economic activity increased air quality worldwide [35]. The change was dramatic in developed nations such as Europe and the US [36]. A reduction in NO_2_, SO_2_, and PM was observed during the lockdown process due to strict lockdowns in most affected countries. During the COVID-19 lockdown time, the concentrations decreased by more than half. However, the reduction achieved is not expected to be maintainable [37].

According to some studies, the COVID-19 pandemic has raised emissions compared to last year [38,39]. However, a lower PM concentration in some Western European cities is less significant since the residential heating system was the main contributor to PM [40]. There is also evidence that PM concentrations increased during the lockdown phase. This was attributed to increased domestic heating and industrial activity in peripheral regions and some areas of northeast China, thereby compensating for the disruption of manufacturing activities in major cities [41]. PM concentration can also increase due to the long-term transport phenomena of PM from adjacent agricultural and industrial zones, as demonstrated in Brazil and Morocco [42,43]. Additionally, these studies indicate that traffic-related policy interventions are inadequate to resolve air quality issues, and other relevant departments must be taken into account [41]. Furthermore, essential steps are needed concerning agricultural burning or the search for ideal sites for industrial activities.

Black carbon (BC) concentrations were higher all day in the pre-COVID stage than in other stages. Meanwhile, BC concentrations had few variations between lockdown, secondary, and tertiary reaction cycles, indicating a significant source of BC in Suzhou’s industrial processes. Persistent precipitation triggered the lower Spring Festival and tertiary response concentrations of BC. PM pollution builds rapidly at high levels under static weather situations and then experiences cross-border transportation processes, resulting in complex health and environmental consequences [44,45,46,47]. In addition, air pollution has dynamic relationships with widespread climate and weather [45,48,49]. Li et al. [50] found that during the COVID-19 in China’s Yangtze River Delta (YRD), human activities—industrial operations, travel vehicles, operating buildings, etc.—were substantially reduced, resulting in lower PM_2.5_ emissions of up to 27–46%.

A study described a higher concentration of organic carbon (OC) in PM_1.8_ and PM_2.5_ in winter compared to summer [51]. The researchers further explained that a colder and stable environment always favors newly formed organic substances condensing from vehicular emission [2,52]. In addition, an apparent seasonal change in PAHs has also been reported [51], with a higher and lower level in the winter and summer seasons, respectively. According to this research, more biomass burning occurs in winter, and the lower temperature favors less volatility and increases the gas conversion rate into PM-bound particles of PAHs.

### 2.2. Inference of Lockdown on the Primary and Secondary Formation of PM

Overall, there have been major reductions in PM formation in some cities of China [53] but no evidence of a decrease in PM concentration in European countries and the US [36,40]. This is because non-transportation sources, which include domestic heating, biomass burning, and food cooking, contribute significantly to aerosol concentration in some contexts [36,40]. The concentrations of PM_10_ and PM_2.5_ were 36.5 and 35.9 μg/m^3^ in Suzhou during lockdown, lower than the pre-COVID concentrations of 37.2% and 38.3%, respectively, although the daily variance of PM during lockdown corresponded to its pre-COVID variance, irrespective of the substantial drop [54]. During the lockdown in many major cities worldwide, air pollutants decreased dramatically. Studies have shown that the lockdown syndrome attributed to COVID-19 has influenced the mechanism of primary and secondary particulate matter formation [54]. In addition, the findings show that travel restrictions have, in most cases, significantly decreased NO_2_ and CO contaminants directly connected with the transport sector [35,42,55].

On average, NO_2_ concentrations in Barcelona and Madrid exhibited a 50% and 62% decline in March 2020, respectively, compared to the 2019 results. However, these reductions have not been recorded in American cities like New York and Memphis [56,57]. It could also show that the pollution caused by traffic in these cities is small [56]. In comparison, many investigations of Brazilian, Chinese, and South Asian cities show that greening the transport sector will offer significant advantages in terms of air quality excellence [42,58,59]. Compared to changes in SO_2_, NO_2,__,_ and CO during the lockdown, O_3_ concentrations were significantly enhanced due to the sharp decrease in NOx. An increased concentration of O_3_ as an atmospheric oxidant can increase the formation rate of secondary organic and inorganic PM. Significant declines in transport NOx emissions during the lockdown were also reported by Huang et al. [60], which encouraged the production of secondary particulates caused by elevated ozone levels and night NO_3_ radical production during night lockdown. Officials should also be mindful that steps to reduce such contaminants, such as NO_2_ and PM, may raise the concentration of secondary pollutants, such as ground-level ozone, and trigger other health problems. However, more research is required to better identify the primary reaction mechanisms and the implications of other atmospheric influences [61].

These regulatory steps have significantly reduced primary emissions of PM, while secondary pollutant like ozone (O_3_) is still prominent [62]. In addition, many investigations have revealed that complex air pollution has come from primary industrial pollutants, traffic, heating processes, and power plants, while secondary pollutants are produced by complex chemical, biological, and physical processes [47,63,64,65,66,67].

### 2.3. Influence of Lockdown on the Composition of PM

**Figure 1:**
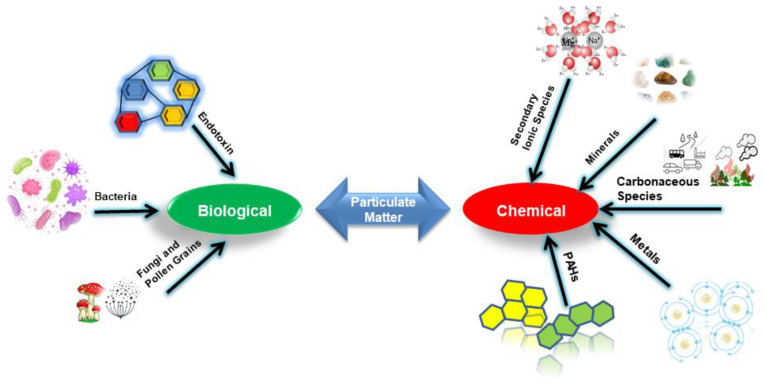
Composition of the different chemical and biological components of PM.

Ambient PM consists of various biological and chemical components [68]. The chemical constituents of PM include minerals (metal oxides), secondary inorganic PM, rare earth metals, elemental carbon (EC), sea salt and organic matter, water-soluble ions, rare earth metals, organic constitutes (e.g., PAHs, OC, organic matter, and volatile organic compounds (VOCs)), inorganic constituents, inorganic secondary aerosol, marine salt, and trace elements [2]. From these PAHs, secondary inorganic species described as the main components of PM, such as nitrate, sulfate, ammonia, and carbonaceous species (OC, EC), are of great concern due to their toxicity and carcinogenicity [69]. Figure 1 shows different chemical and biological constituents of PM. The PM components with a biological origin are termed bioaerosols OC, and are included in a similar category in some studies [70,71]. These bacteria, pollens, and plant-related fragments are usually found in coarse PM [72]. However, some bacterial and fungal spores were also reported in fine PM [73]. These tend to attach to coarser particulate fractions.

Ambient PM contains diverse chemical elements such as carbonaceous, elemental, and organic substances. The individual concentration of these components forms 10 to 30% of the total mass of PM [74,75]. They are highly variable in concentration, depending upon source emission, meteorological conditions, and other factors [76]. The following are the main chemical species present in PM:

During the lockdown in several major cities, air pollutants decreased significantly. For example, PM_2.5_, PM_10_, and BC concentration in Suzhou was recorded at 37%, 38%, and 53% less during lockdown than in the pre-COVID period, respectively [54]; while in Wuhan, PM_2.5_ level decreased by 41% and PM_10_ by 33% [77]. In Delhi, during the lockdown phases, PM_10_, PM_2.5,_ and BC decreased by about 52%, 53%, and 78%, respectively, compared to the pre-lockdown period [78,79]. In Washington, PM_2.5_ and BC concentrations decreased by 33% and 25% during lockdown implementations [80]. A brief description of lockdown impacts on PM provided in Table 1 can be essential to demonstrate how lockdown amid COVID-19 modulated overall pollution in different megacities. Likewise, comparing Table 1 and Table 2 with Table 3 would be helpful for understanding lockdown-based air pollution, the impact of meteorological attributes, and corresponding changes in reported cases of COVID-19.

Concentrations of water-soluble ions (WSI) were 58.6% less than in the pre-COVID period. In addition, the PM_2.5_ and ion ratios showed the lowest lockdown values, up to 27.4%. This disparity demonstrated the significant changes during the lockdown in the chemical composition of PM_2.5_. Specifically, during the lockdown actions, as per Zheng et al. [96], primary emissions declined while secondary production of PM_2.5_ increased, resulting in less total mass concentration of PM_2.5_ and different chemical composition. According to Sun et al. [97], 25–46% of all gaseous species (NO_2_, SO_2_, and CO) were decreased, with a 30% to 50% reduction in aerosol form (fossil-fuel related PM, predominantly from coal combustion emissions, cooking-related organic PM, and biomass-burning organic aerosol) due to Chinese New Year. Through the lockdown period in Suzhou, the ionic arrangement, in order of concentration, was NO_3_ > NH_4_ > SO_4_^2−^ > Cl^−^> Ca^2+^ > K > Mg^2^ > Na^+^; while during the pre-COVID phase, they were rearranged into NO_3_ > SO_4_^2−^ > NH_4_ > Cl > Ca^2+^ > K > Na^+^ > Mg^2+^. Compared with the pre-COVID ion levels, it was reported that ions NO^3−^, NH^4+^-, SO_4_^2−^, Cl^−^, Ca^2+^, K^+^, and Na^+^ dropped by 66.3, 48.8, 52.9, 57.9, and 76.3 in terms of percentage concentrations, respectively, in the lockdown period. At the same time, Mg^2+^ exhibited an increase of 30.2% [54]. Overall, compared to the pre-COVID time, during the lockdown in Suzhou, the PM_10_, PM_2.5_, BC, and WSIs decreased by 38.3, 37.2, 53.3, and 58.6%, respectively [54].

Furthermore, most research on air pollution “lockdown” focuses on “classic” contaminants such as NO_2_, CO, SO_2_, PM_2.5_, and PM_10_ [98,99,100,101]. Ivana et al. [102] reported a 35% decrease in NO_2_ and PM_1_, alongside a 26% decrease in total PAHs, near road traffic measuring sites. Only the concentration of NO_2_ decreased marginally at the residential measurement site; PM_1_ and PAHs levels were comparable to the previous year. Zhang et al. [103] found that PAH concentrations decreased 52.6%, 36.6%, and 36.7% from February to April of 2020 relative to the same time in the previous year. The changes in northern China are consistent with a decrease in SO_2_ and NO_2_ that grew during COVID-19 control and moderated a bit after the lockdown was lifted. In addition, the composition of PAHs in Kanazawa University Wajima Air Monitoring Station (KUWAMS) changed little before, during, and compared to previous years in the COVID-19 outbreak, indicating a stable source composition. These findings emphasize the importance of reducing the emission intensity in China for reducing PAH transport over long distances and pollution levels in downwind areas.

### 2.4. Influence of Lockdown on PM_2.5−_ and PM_10−_ Based Air Quality Index

**Figure 2:**
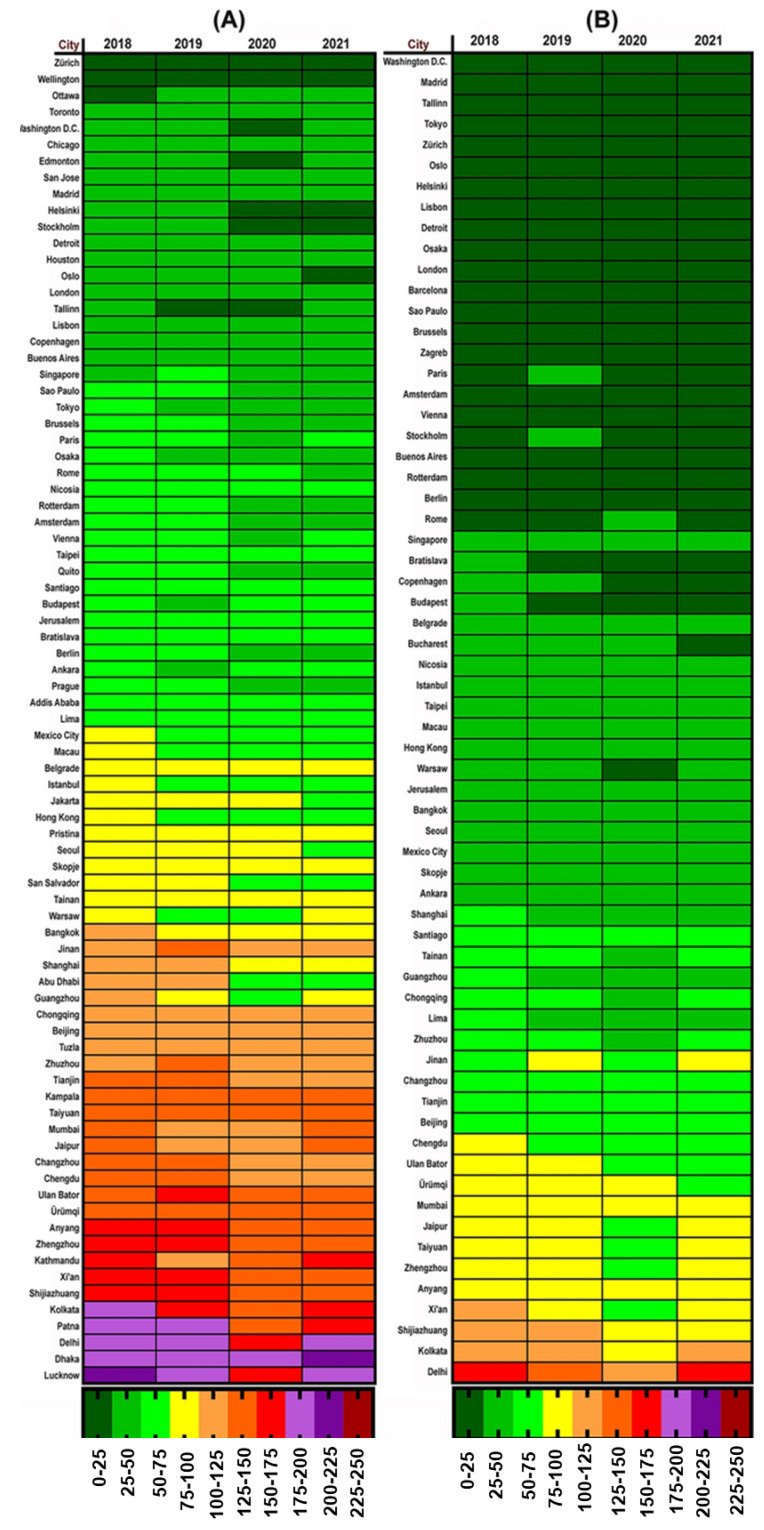
Heat map created with GraphPad Prism showing average medians of the four first months’ AQIs following 2020 lockdown and comparisons with 2018, 2019, and 2021. (**A**) AQI–PM_2.5_ variation, (**B**) AQI–PM_10_ variation. Reproduced from Ref. [104] with permission. Copyright, 2022, Springer Nature.

Sarmadi and his colleagues [104] studied variations in the AQI during the first four months of each year (from 2018–2021), evaluating the AQI from 87 industrialized, polluted, and highly populated metropolises in 57 countries. Noticeably, of these 87 metropolises, 58 were capital cities, while the remainder were among the world’s top 100 heavily polluted and industrialized cities. As shown in Figure 2, the cities with the lowest PM_2.5_ and PM_10_ AQI values were Edmonton, Washington, Zurich, and Tallinn, with corresponding AQI of 0.10, 0.18, 2.31, and 3.98. Meanwhile, in 2020, the highest AQI levels were in Dhaka, Delhi, Ulaanbaatar, Seoul, and Jerusalem, with AQIs of 182.18, 106.36, 11.19, 26.86, and 36.62, respectively.

**Figure 3:**
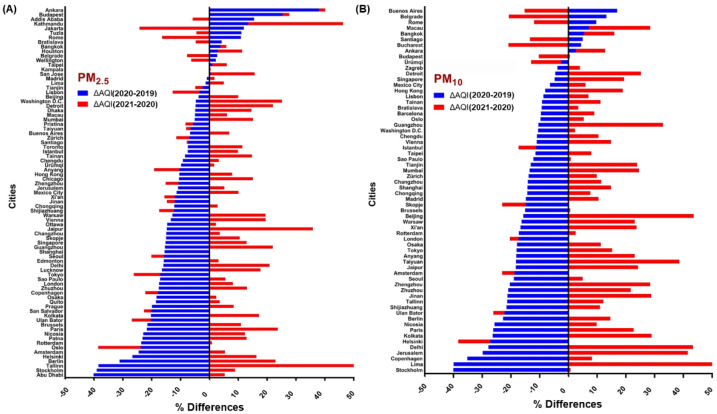
Percentage change of AQI PM_2.5_, PM_10_, NO_2,_, and SO_2_ between 2020—during the lockdown period—2019, and 2021, in different cities worldwide. (**A**) AQI–PM_2.5_ variation, **(B**) AQI–PM_10_ variation. Reproduced from Ref. [104] with permission. Copyright, Copyright, 2022, Springer Nature.

According to AQI, during the first quarter of each year, shown in Figure 3, the AQI in 2020 improved significantly in most cities compared to pre-COVID (2019) time; however, most of the metropolises regained poor AQI scores in 2021. Similar trends were observed in other lockdown-impacted AQI assessment studies [105,106]. The greatest percentage decrease in PM_2.5_ and PM_10_ in 2020 compared to 2019 was seen in places such as Stockholm and Abu Dhabi (−40.05% and −40.13%), while the greatest increases were seen in Ankara and Buenos Aires (+37.97% and +16.95%, respectively). In countries with a +ve variance percentage, the AQI increased as well as dropped over time. Only 13% (7 of 55) and 25% (17 of 67) of cities with smaller AQI–PM_10_ and AQI–PM_2.5_ values in 2020 than in 2019 showed a declining trend in 2021, respectively, while AQI values rose in some other cities in 2021.

**Figure 4:**
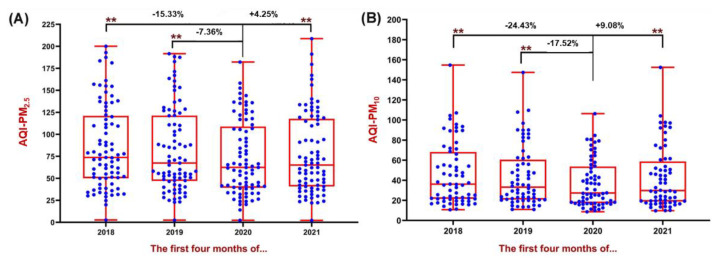
Box and whiskers plot depicting AQI variations for 87 cities in the world. The data shows the average for the first four months of 2018–2021 (January 1st–April 30th). (**A**) AQI–PM_2.5_ variation, (**B**) AQI–PM_10_ variation. ** *p* < 0.01, NA > 0.05. Reproduced from Ref. [104] with permission. Copyright, Copyright, 2022, Springer Nature.

The mean AQI–PM_2.5_ in 2020 declined by 7% and 15%, respectively, when compared with 2019 and 2018, and the mean AQI–PM_10_ decreased by 18% and 24%, showing a better AQI, attributed to a decline in PM (Figure 4A,B). It’s worth noting that those same stations measuring AQI may be situated near major roadways and airports, where PM levels are likely to be elevated [107].

## 3. Influence of Meteorological Factors on PM Level and COVID-19 Cases

Metrological attributes are the most influential factors affecting ambient PM concentration. In addition, various meteorological factors such as precipitation, temperature, wind speed, RH, and dispersion of ambient PM play a vital role in their life cycle and persistence [108]. Therefore, the statistical analysis of PM and COVID-19 with meteorological factors is considered helpful in understanding emission sources and effectively managing PM-linked COVID-19 pollution.

Zhao et al. [109] also reported meteorological factors influencing carbonaceous species (EC, OC, primary organic compounds (POC), and secondary organic compounds (SOC)) and found an increasing trend in winter and autumn and a lower influence in the summer season. Further, they reported that SOC increased more than POC in winter. An increase in SOC was found more than POC in winter. A similar higher SOC trend was observed in winter during a study conducted in several cities in China [110]. Generally, stable atmospheric conditions with lower temperatures, primarily occurring in winter and autumn, favor the accumulation of PM, accelerate the adsorption of VOC on existing material, and increase the condensation process [2,111]. Compared with secondary inorganic ions, the levels of SOC showed different seasonal trends. In Suzhou, the BC concentrations were higher during the pre-COVID stage than during the lockdown period, but the decrease was mainly due to continuing precipitation [54]. Precipitation also reduces the ambient PM and associated species by washing out the atmospheric PM [112].

Similarly, in the USA, rainfall is linked negatively and weakly to COVID-19 [113]. In Italy, however, rainfall increases the transmission of diseases with every average inch per day. Another study discovered that the number of cases per day has risen by 56.10 [114], possibly due to surface pollution that has led to COVID-19 spreading rapidly.

Pateraki et al. [115] investigated the interaction of different-sized PM and meteorological attributes and suggested that the increase in secondary PM is linked with an increase in temperature. Fine PM transforms in the presence of solar radiation, which is higher in the warmer season. Similar behavior was found with other PM fractions. Generally, PM_2.5_ makes up about 50% of the total PM_10_ fraction, from which most of the fine PM comprises SO_4_^−2^ and NO^3−^. The higher sulfate level is a favorable photochemical condition that encourages sulfate formation and inhibits nitrate’s condensation process. The elevated level of SO_4_^2−^ ions suggests the increased concentration of PM_10_ has a relation to an increase in temperature. Pateraki et al. [111] noticed the increments in the concentrations of PM_2.5_ and PM_10_ were greater on days with higher temperatures. They further reported that in temperatures up to 21.7 °C, secondary particle generation occurs along with the increase in PM_10_.

On the other hand, COVID-19 showed a negative trend with an increase in temperature in the US. When the minimum and average temperature increases substantially, it lowers the number of cases of COVID-19 [113]. An asymmetric nexus was observed in China between temperature and COVID-19 patients. Some were positive, a few came up with negative, and some observed mixed trends [116]. In another study, a temperature rise was not significant in the containment or minimization of COVID-19 infections [117]. However, Liu et al. [118] found a reduction in the cause (as with the USA), with a 1 ℃ rise in air temperature correlated with a decrease in daily reported case numbers. According to another study, lower and higher temperatures may help reduce the incidence of COVID-19 [119]. In Italy, when the average daily temperature rose by 1 ℉, the number of cases per day decreased by 6.4, similar to findings from studies in China and the USA [114].

Humidity is another major meteorological factor which highly related to PM concentration. Pateraki et al. [115] reported a negative effect of humidity on the increment of PM, i.e., with an increase in humidity, the PM_10_ and PM_2.5_ were reduced. However, change in moisture does not affect the number of COVID-19 cases in the USA [113]. Though absolute humidity (AH) was closely related in China, 1 g/m^3^ AH increases were significantly correlated with a reported reduced event [118]. Similarly, many studies proposed a negative relationship between wind speed and PAH levels [120,121]. Wind always dilutes the air, and the PM concentration declines [122]. However, wind speed is insignificant in virus spread [113].

Table 2 depicts significant studies which addressed various interactions among different meteorological factors, PM, and COVID-19 conditions in different countries.

**Table 2:** Meteorological factors’ effects on COVID-19 and PM pollution.

Meteorological Factor	Location	COVID-19 or PM Pollution	Findings	Reference
Temperature	USA (New York)	COVID-19	COVID-19 cases decreased significantly with an increase in average and minimum temperatures.	[113]
Temperature	China (10 affected provinces)	COVID-19	Temperature and COVID-19: asymmetric nexus—some show positive, some show negative, and a few show mixed signs.	[123]
Temperature	China (Wuhan)	COVID-19	A temperature increase does not appear to be able to slow down or contain COVID-19 infections.	[117]
Temperature	China (17 different cities)	COVID-19	An increase of 1 ℃ in the ambient temperature was associated with a decline in the daily confirmed case count.	[118]
Temperature	China	COVID-19	Lower and higher temperatures may reduce COVID-19 incidence.	[119]
Temperature	Italy	COVID-19	With an increase of 1 °C in average daily temperature, the number of cases decreased by approximately 6.4 per day.	[114]
Temperature	India	COVID-19	Temperature causes an increase in the number of daily infections, and co-variability accounts for 85–50% of them.	[124]
Temperature	India	COVID-19	A positive correlation between new cases of COVID-19 and the increasing temperature in the region.	[125]
Temperature	India	PM	Temperature and PM_2.5_ showed a strong negative correlation (r = −0.546).	[83]
Temperature	India’s 9 most affected cities	PM	The diurnal range in temperature is not significantly correlated.	[126]
Temperature	Top 20 countries	COVID-19	The number of confirmed cases and deaths associated with COVID-19 decreases with high temperatures and increases with cold temperatures.	[127]
Humidity	USA (New York)	COVID-19	Humidity doesn’t seem to play a significant role in the total number of cases.	[113]
Humidity	China (all provincial capitals)	COVID-19	An increase of 1 g/m^3^ in absolute humidity was significantly associated with a reduction in confirmed cases.	[118]
Humidity	China	COVID-19	The incidence of COVID-19 and absolute humidity did not show a significant association.	[119]
Humidity	India, 12 cities	COVID-19	No correlation with RH.	[124]
Humidity	India	COVID-19	COVID-19 shows a negative association with RH values up to mid-May, and then shows a positive association (showing again that increasing humidity does not affect India’s COVID-19 rates).	[125]
Humidity	India’s 9 most affected cities	COVID-19	The daily range of RH is not significantly correlated.	[126]
Humidity	Pakistan	COVID-19	Except for Lahore (r = 0.175), there is a significant correlation between COVID-19 cases and humidity.	[128]
Humidity	Top 20 countries	COVID-19	There is a strong correlation between RH and COVID-19 incidence. RH increases the viability and persistence of the virus. Low RH is reported to prolong the viability and stability of Coronaviruses on contaminated surfaces.	[127]
Humidity	Iran (Tehran, Mazandaran, Alborz, Gilan, and Qom)	COVID-19	COVID-19 cases increased with RH.	[129]
Rain Fall	USA	COVID-19	COVID-19 is negatively and weakly correlated.	[113]
Rain Fall	Italy	COVID-19	Each inch/day increases disease transmission.	[114]
Rain Fall	India	PM	Amount of rainfall contributed to the reduction in PM.	[82]
Wind speed	USA	COVID-19	The speed of the wind does not play a significant role in the spread of viruses.	[113]
Air masses’ movement	India	PM	The movement of air masses also played a significant role in reducing PM.	[82]
Wind speed and pressure	Top 20 countries	COVID-19	Virus spread is accelerated by both wind speed and surface pressure intensities.	[127]
Wind speed,	Iran (Tehran, Mazandaran, Alborz, Gilan, and Qom)	COVID-19	COVID-19 cases increased due to the low wind speed.	[129]
Radiation exposure	Iran (Tehran, Mazandaran, Alborz, Gilan, and Qom)	COVID-19	COVID-19 increased with high solar radiation.	[129]

## 4. Health Implications Due to co-Exposure to PM and COVID-19

**Figure 5:**
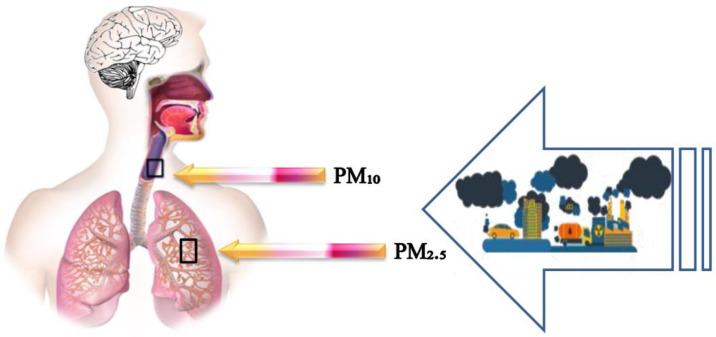
Comparison of breathing penetration of PM_2.5_ and PM_10_ into the human lungs.

Prior epidemiological research has shown an important relationship between exposure to outdoor pollutants and lung disease and heart disorders [130,131]. Moreover, the toxicity of PM is directly related to its size [132]. Fine-fraction PM_2.5_ is relatively more persistent in the atmosphere and can easily be moved into the human body by air (Figure 5). Exposure to PM_2.5_ can reduce life expectancy by 5.5 years [133].

In China, Zhu et al. [31] examined the association of PM_2.5_, PM_10,_ and other contaminants in 120 cities and reported COVID-19 daily cases. Significant positive associations of these contaminants with reported COVID-19 cases have been identified. The findings of this study support that COVID-19 infection can be caused by ambient air pollution. In contrast, in the health emergency in Lombardy (Italy) several days earlier, from 10 February to 27 March 2020, Bontempi [32] first analyzed the PM_10_ situation. The data on PM_10_ levels and infection cases analyzed in Piedmont and Lombardy revealed clear associations between high PM_10_ levels and COVID-19 virus transmission. Assuming that the transport effects of PM_10_ enabled the spread of the virus in Lombardy would be an improper health risk evaluation. The results of prolonged exposure to air contaminants in Italy [30] and the US [26,27] suggest an obstruction of recovery, leading to severe and more lethal types of disease.

In that regard, another study explored COVID-19 environmental transmission dynamics mechanisms for a potential approach to cope with future coronavirus-like epidemics. The results showed that two mechanisms in a particular environment triggered accelerated COVID-19 transmission dynamism: air pollution-to-human spread and human-to-human spread in a high population density setting. The two key results were (i) the dynamics of COVID-19 in the northern region of Italy are highly connected with air pollution in cities; and (ii) towns with more than 100 polluted days (having higher levels than the PM_10_ standard) showed an exceptionally higher number of infected cases (approximately 3340 people), while cities with less than 100 polluted days exhibited an average infection rate [134,135]. Moreover, Sanità di Toppi et al. [136] hypothesized that the COVID-19 virus might use a “highway” of atmospheric PM to facilitate its indirect diffusion. The authors suggested that this question requires a more immediate and comprehensive study. Based on the findings from many recent articles, we completely support this scientific hypothesis. Finally, the COVID-19 pandemic has paradoxically decreased the number of deaths in quarantine due to the massive reduction in air pollution, which significantly reduces the number of deaths caused by air pollution itself [33].

In addition to previous facts, air pollution mitigation will help control the spread of the pandemic and improve the coping ability of sick persons. Moreover, several studies have found strong relationships between COVID-19 transmission/mortality and elevated environmental pollution [134,137]. Research on Italian regions indicated higher air pollution spread rates in northern areas [30]. Furthermore, exposure to long-term pollution will indirectly escalate susceptibility to COVID-19 by impacting the respiratory system [30,36]. Improving air quality would also help tackle short- and long-term problems related to COVID-19 and other pandemics.

**Figure 6:**
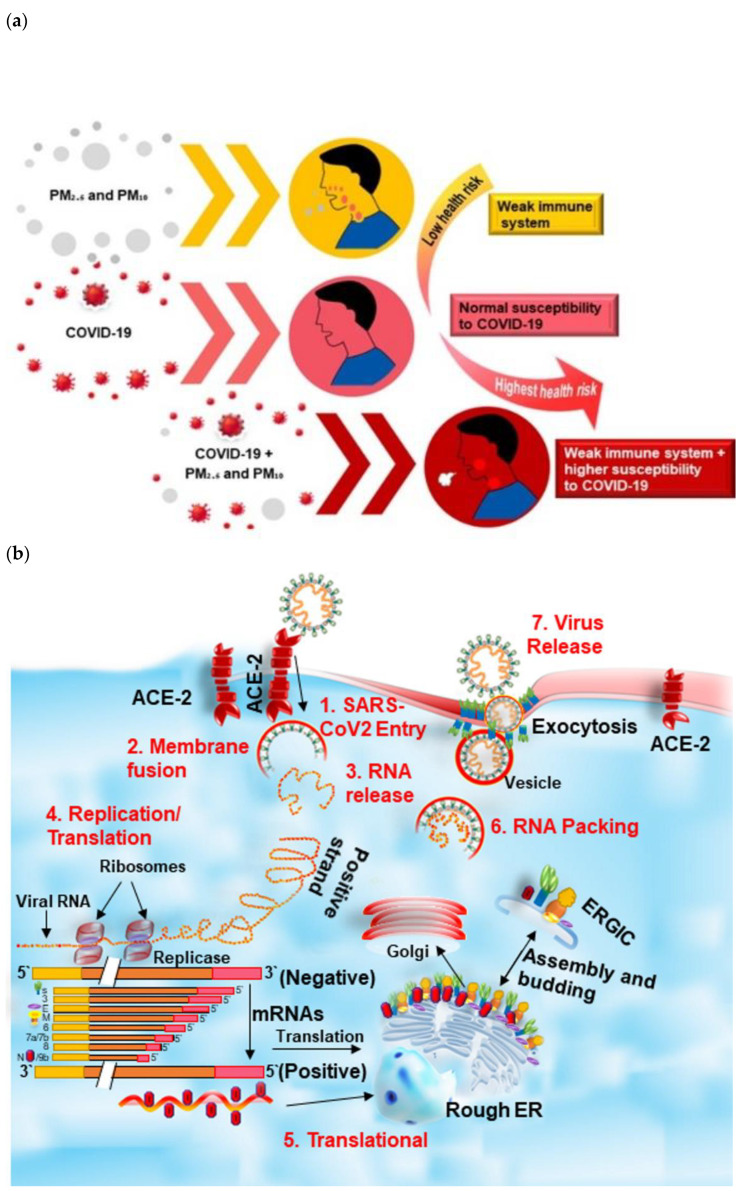
(**a**) COVID-19 health risk in the presence and absence of PM pollution; (**b**) the process of SARS-CoV-2 pathogenicity based on replication attachment.

Moreover, the viruses will live longer in a linear relationship and become more violent in an immune state. Individuals who live in more polluted areas are relatively more vulnerable to respiratory disorders [20] and are more exposed to viral sickness [21]. Continuous pollution inhalation damages the first protection spot’s upper airways, primarily the cilia [29]. Furthermore, the COVID-19 pandemic death toll may have reduced during this period because healthy air significantly reduced the deaths caused by air pollution itself [33]. The poorer pre-health conditions caused by air pollution tended to be associated with more COVID-19 deaths in Lombardy and Emilia Romagna. Higher air pollution levels in Northern Italy have been found to be an additional factor for this region’s high lethality [30]. The statement that PM_10_ transport effects facilitated Lombardy’s virus diffusion would be an invalid health risk assessment. On the other hand, it has been demonstrated that air pollution increases COVID-19 susceptibility [138]. A recent study showed that the relationship between COVID-19 and PM (PM_10_ and PM_2.5_) was positive and significant [139]. Based on the findings related to PM and COVID-19, the possible health risk level of COVID-19 in the presence and absence of PM pollution is depicted in Figure 6a.

### PM and COVID-19 Mechanism Inside the Human Body

The function and diversity of the normal microbiome are essential for the host’s health. Although the impact of PM on human health is well known, the role of infectious particles in bacterial ecosystems was ignored [140]. BC, which is a major cause of pneumonia in respiratory infectious diseases, plays a big role in the risk of acquiring infectious respiratory diseases and changes the structure and function of the biofilms of both types of pneumonia (*Staphylococcus aureus* and *Streptococcus pneumonia*) [141]. Evidence indicates that outdoor and indoor dust modifies opportunistic pathogenic agents’ virulence, production, and biofilm in microbial growth. The exposure to gradually growing indoor and outdoor dust concentrations of three opportunistic bacteria (*Escherichia coli*, *Enterococcus faecalis,* and *Pseudomonas aeruginosa*) have shown variance growth trends. This correlates with the increased formation of biofilm and oxidative stress exposure following the hydrogen peroxide challenge [142,143].

All mutations of coronaviruses contain unique viral reproduction genes, nucleocapsid, and spikes in downstream regions of the open reading frame gene (ORF1) [144]. In addition, the glycoprotein spikes on the coronavirus’ external surface are responsible for the virus’ attachment to host cells (Figure 6b). The receptor-binding domain (RBD) is loosely connected to the virus’ surface and allows it to infect multiple hosts [145,146]. Other coronaviruses recognize carbohydrates or aminopeptidases as a principal receptor for entry into cells of humans, whereas exopeptidases are recognized in SARS-CoV and MERS-CoV [147]. The coronavirus input protocol relies on cell proteases such as HAT (human airway trypsin-like protease), cathepsin, and TMPRSS2 (transmembrane protease, serine 2), which breaks the spike protein and alters its pervasiveness [148,149]. MERS-coronavirus use the DPP4 (dipeptidyl peptidase 4), while ACE2 (angiotensin-converting enzyme 2) is required as the main receptor by HCoV-NL63 and SARS-coronavirus [146,147]. The SARS-CoV-2 virus consists of the typical spike protein instead of the usual spike protein design. In addition, it includes all the polyproteins, nucleoproteins, and membrane proteins found in the virus, such as RNA polymerase, papain-like protease, 3–chymotrypsin-like protease, glycoprotein, helicase, and accessory proteins. [150,151]. The SARS-CoV-2 spike protein contains a 3-D structure in the RBD region for van der Waals [152]. In the RBD area of SARS-CoV-2, 394 glutamine residues are detected by the essential residue lysine 31 on the human ACE2 receptor [153]. The entire pathogenicity process of SARS-CoV-2, from replication to attachment, is well described in Figure 6b.

## 5. Health Risk Assessment Due to the Combination of PM and COVID-19

Ambient PM has been linked to increased respiratory morbidity and mortality [154], particularly in vulnerable persons, and was associated with cardiorespiratory events such as asthma, pulmonary obstruction, and thrombosis [155,156]. Setti et al. [157] have recently quantified the first preliminaries to the effect that SARS-CoV-2 can occur on ambient PM, indicating that it might represent a possible initial indicator of COVID-19 under circumstances of atmospheric constancy and elevated PM levels. However, the research does not provide details regarding the progression or severity of COVID-19. In vivo and in vitro studies showed PM’s involvement in exacerbating viral respiratory infections [158]. In vitro studies indicate that BC, the main factor of pneumonia in the body, is highly affected by infectious respiratory disease predisposition. VOCs are mainly indoor contaminants and contain benzene, xylene, toluene, terpenes, and PAHs. Formaldehyde is produced by the reaction between terpenes and NOx or ozone in an indoor environment. Formaldehyde is generally categorized as a greater risk for nasopharyngeal carcinoma and leukemia.

### Variation in COVID-19 Cases with Ambient PM_2.5_ and PM_10_ Level

There is also a clear correlation between concentrations of PM_2.5_ [12,26,27,31,113,159,160,161,162,163,164,165], PM_10_ [113,134,135,160], and COVID-19 cases, as shown in Table 3. The first evidence of the temporal connection between COVID-19 and air pollution was recorded in China [31].

**Table 3:** PM pollution and COVID-19 association.

Location	Period	Aim	Effect	Data Analysis	Reference
USA (3000 counties)	Data up to 22 April 2020	Estimation of long-term COVID-19 deaths based on average exposure to PM_2.5_.	A 1 μg/m^3^ increase in PM_2.5_ caused an 8% increase in the COVID-19 death rate.	Zero-inflated negative binomial models	[26]
US (3089 counties)	Data up to 18 June 2020	COVID-19 death rates outcome and long-term average PM_2.5_ exposure.	A 1 μg/m^3^ rise in PM_2.5_ concentration was associated with an 11% increase in COVID-19 mortalities.	Negative binomial mixed model	[27]
USA (California)	From 4 March to 24 April 2020	PM_2.5_, PM_10_, and NO_2_ pollution association with confirmed cases.	PM_2.5_: Kendall r (−0.359); Spearman r (−0.453) PM_10_: Kendall r (−0.287); Spearman r (−0.375). Significant correlation.	Spearman and Kendall correlation tests	[113]
Queens County, New York (U.S.A)	From 1 March to 20 April 2020	Association between daily confirmed cases, total deaths and PM_2.5_.	Daily cases association = −0.4029 (CI %: 0.6478–0.6896); mortality association = −0.1151 (CI%: 0.7966–0.9971).	Negative binomial regression model	[159]
China (120 cities)	From 23 Jan to 29 February 2020	The relationship between daily confirmed cases and air pollution (PM_2.5_, PM_10_, and NO_2_) over time.	PM_2.5_: 10 μg/m^3^ increase (lag 0–14) was associated with a 2.24% increase in daily new confirmed cases; PM_10_: a 10 μg/m^3^ increase (lag 0–14) was associated with a 1.76% increase in daily confirmed new cases.	Generalized additive model (GAM)	[31]
Wuhan, Xiaogan, and Huanggang (China)	From 25 Jan to 29 February 2020	PM_2.5_, PM_10_, and NO_2_ pollution and daily confirmed cases temporal association.	PM_2.5_: Wuhan (RR = 1.036, CI:1.032–1.039); Xiaogan (RR = 1.059, CI = 1.046–1.072); Huanggang (RR = 1.144, CI = 1.12–1.169) PM_10_: Wuhan (RR = 0.964, CI: 0.961–0.967); Xiaogan (RR = 0.961, CI = 0.950–0.972); Huanggang (RR = 0.915, CI = 0.896–0.934).	Multivariate Poisson regression	[161]
Wuhan and Xiaogan	From 26 Jan to 29 February 2020	Daily confirmed cases and air pollution PM_2.5_, PM_10_, and NO_2_ relation.	PM_2.5_: Wuhan (R^2^ = 0.174, *p* < 0.05); Xiaogan (R^2^ = 0.23, *p* < 0.01). PM_10_: Wuhan (R^2^ = 0.105; *p* > 0.05); Xiaogan (R^2^ = 0.158, *p* < 0.05).	Simple linear regression	[162]
49 cities of China	Data up to March 22, 2020	Relationship between air pollution level (PM_2.5_ and PM_10_) and fatality rate.	PM_2.5_: a 10 μg/m^3^ increase in PM_2.5_ was associated with a 0.24% (0.01%–0.48%) increase in fatality rate; PM_10_: 10 μg/m^3^ increase in PM_10_ was associated with a 0.26% (0.00%–0.51%) increase in fatality rate.	Multiple linear regression	[164]
Milan (Italy)	From 1 Jan to 30 April 2020	PM_2.5_ and PM_10_ and total deaths (total cases, daily confirmed cases) association over time.	PM_2.5_: R = −0.39; R = 0.25; R = −0.53; PM_10_: R = −0.30; R = 0.35; R = −0.49.	Pearson coefficient correlation	[165]
7 provinces of Lombardy, Italy; 6 provinces of Piedmont, Italy	From 10 February to 12 March 2020	Spatial description of PM_10_ exceedances versus COVID-19 cases.	Lombardy: PM_10_ exceeding between 0 and 8, COVID-19 incidence % between 0.03 and 0.49; Piedmont: PM_10_ exceeding between 3 and 12, COVID-19 incidence % between 0.01 and 0.03.	Descriptive analysis	[32]
55 Italian Provinces	Data up to April 7, 2020	The relationship between confirmed cases and PM_10_.	COVID-19 in Northern Italy is highly correlated with air pollution levels measured in cities with days exceeding PM_10_ limits.	Hierarchical multiple regression model	[135]
71 Italian provinces	Data up to 27 April 2020	Air pollution levels (PM_2.5_, PM_10_, NO_2_) and total confirmed cases.	PM_2.5_: R^2^ = 0.340, *p* < 0.01; PM_10_: R^2^ = 0.267, *p* < 0.01.	Pearson regression coefficient analysis	[160]
110 Italian provinces	From 24 February to 13 March 2020	PM_10_ concentration exceedance relation with spreading of COVID-19 infection.	Daily PM_10_ exceedances and spreading of COVID-19 infection in 110 Italian provinces are geographically linked.	Pearson’s coefficient utilized for correlation analysis	[166]
Pakistan			COVID-19 cases were significantly correlated with PM_2.5_ and climatic factors at *p* < 0.05, except for Lahore.		[128]
Global (27 countries, including China, India, and Europe)	Feb-Mar 2020	Researchers evaluated whether lockdown events reduced air pollution levels by using satellite data and more than 10,000 air quality stations.	Over 2 weeks following the lockdown, 7400 premature deaths (340 to 14,600) and 6600 (4900 to 7900) pediatric asthma cases were avoided. As a result of avoiding PM_2.5_ exposure, China avoided 1400 premature deaths (1100–1700) and India avoided 5300 (1000–11700). Assuming the lockdown-induced reduction in concentrations persists throughout 2020, 0.78 (0.09–1.5) million premature deaths and 1.6 (0.8–2) million pediatric asthma cases could be avoided around the world.		[167]

In 120 Chinese cities, Zhu et al. [31] studied the connection between PM and viral infection caused by the novel coronavirus. Between 23 January 2020, and 29 February 2020, over 58,000 (70%) daily confirmed new cases in China were utilized in research. They employed a general additive model to determine the impact of meteorological factors and ambient pollution on the distribution of COVID-19 by using the moving average method to detect the accumulated environmental pollution lag effect. With a focus on the variables of population density and size, the effect of PM_2.5_ on daily reported cases was concluded to be greater than that of PM_10_. In particular, they observed that the 10 μg/m^3^ rise in PM_2.5_ concentration and PM_10_ (0–14 days lag) was associated with a 2.24% (95% CI: 1.02 to 3.46%) rise in regular counts of COVID-19 cases and a 1.76% (95% CI: 0.89 to 2.63%) rise, respectively.

Furthermore, Jiang et al. [161] studied three of China’s most COVID-19-impacted cities, Wuhan, Huanggang, and Xiaogan, by collecting daily positive cases with atmospheric pollutant data from 25 to 29 January. Through multivariant Poisson regression, the authors revealed a significant temporal relationship between PM_2.5_ increase and COVID-19 cases in Wuhan (RR = 1.04, CI: 1.03–1.04), Huanggang (RR = 1.14, CI = 1.12–1.17), and Xiaogan (RR = 1.06, CI = 1.05–1.07). Similarly, an increase in the incidence of COVID-19 with a rise in concentrations of PM_10_ was observed. Li et al. [162] performed simple linear regression comparing PM_10_ and PM_2.5_ concentrations with COVID-19 in Xiaogan and Wuhan. They noticed that a rise in PM_2.5_ in both municipalities was associated with an increase in the incidence of COVID-19 (Wuhan: R^2^ = 0.174, *p* < 0.05 and Xiao Gan: R^2^ = 0.23, *p* < 0.01).

Yao et al. [164] analyzed the spatial distribution of COVID-19 particulate and case fatality rate (CFR) in 49 cities, including Wuhan. First, it was noted that COVID-19 fatality (National Moran index I = 0.16, *p* < 0.0001) showed a strongly positive global autocorrelation with high CFR clusters in Hubei Province. They improved their findings with a multiple linear regression for different impact alternators and confusing variables, such as relative humidity (RH), temperature (T), per capita (gross domestic product), hospital beds, local spatial indicators’ associated map values, and proportion of persons over 65 years old. It was observed that CFR rose by 0.24% (0.01–0.48%) and 0.26% (0.00–0.51%), with the average PM_2.5_ and PM_10_ concentrations increasing by 0.61% (0.09–0.12%) and 0.33% (0.03–0.64%), each with an increment of 10 μg/m^3^, in the 2015 average of PM_2.5_ and PM_10_, respectively.

In addition,, a few researchers have established the association between COVID-19 and environmental contamination in Italy, the world’s second-most affected country at the beginning of the pandemic. In Italy, on 28th July, around 245,000 confirmed cases and 35,107 deaths were reported [168], most of them distributed in the northern regions of Italy, particularly in the Lombardy region. This region is recognized as one of Europe’s most polluted air zones, in which 302 deaths per year (or 13 per 100,000 inhabitants) were attributable to a PM_10_ level that exceeded the WHO standard by 20 μg/m^3^ annually [169].

Bontempi [32] researched two of Northern Italy’s most affected areas, Piedmont and Lombardy. The researcher observed that PM_10_ concentrations were only exceeded a few times in Lombardi cities, most affected at the beginning of the epidemic, on 12th March 2020, based on daily PM_10_ excesses and COVID-19 cases, before the Italian health crisis. Conversely, the COVID-19 incidence was lower in Piedmont cities suffering from heavy PM_10_ concentrations. Researchers concluded that the airborne transmission of COVID-19 and PM_10_ is challenging to establish. Nevertheless, several articles about Northern Italy show that PM, especially PM_2.5_, may play a role in the acceleration and extensive dissemination of COVID-19. Coccia et al. [134] studied the association between air pollution (recording the number of days when the previous year’s PM_10_ concentration was exceeded in some cities) and COVID-19 spread by analyzing data from 55 Italian provincial capitals and infected individuals. On April 7th, 2020, cities that exceeded the previous year’s PM_10_ levels for 100 days or more showed a higher-than-average number of infected persons (approximately 3600 infected persons), while other cities exhibited lower-than-average numbers of infected persons (around 1000 infected persons). Another study of Northern Italy by Frontera et al. [12] showed the function of PM_2.5_ as a contributing factor to the outbreak of COVID-19 by applying Kendall rank and Spearman’s correlation, whether COVID-19 was standardized by population size and whether they conducted regular associations or spatial groups across the country.

Adhikari and Yin [159] studied the COVID-19 and PM_2.5_ relation in Queens County, NY, USA. Data on the daily PM_2.5_ concentration were collected from two terrestrial-monitoring stations, while data on COVID-19 and associated deaths from the US were collected between 1st March and 20th April 2020. They applied a negative binomial regression model on acquired data and considered the cumulative lag impact of PM_2.5_ on COVID-19 confirmation during the last 21 days. They found that PM_2.5_ was significantly related to confirmed new regular cases of COVID-19 (-0.40, CI%: 0.65–0.69) and deaths (-0.12, CI%: 0.80–0.99). Meanwhile, low levels of total PM (average = 4.73 μg/m^3^) in this study area had probably played a less dominant role when infection was reported than in other regions (i.e., Greece), where PM_2.5_ levels reached more than 30 μg/m^3^ per month on average [12,31,160,161].

Researchers have indicated that COVID-19 may have influenced other gas contaminants, such as NO_2_ and SO_2_. Wu et al. [26] analyzed the long-term average exposure to PM_2.5_ and whether it raises the likelihood of COVID-19 fatalities in the US by considering 3000 counties out of 3143 (98 % of the US population). Using exposure modeling, the authors estimated each county’s level of long-term PM_2.5_ exposure, averaged between 2000 and 2016, and death counts of COVID-19 until April 22, 2020. The study results were improved by several complex variables, such as sociodemographic, socioeconomics, behavioral, and meteorologic factors, with zero-inflated negative binomial mixed models. They found that a slight longer-term rise in PM_2.5_ exposure of only 1 μg/m^3^ was related to an 8% (95% CI: 2 to 15%) increase in COVID-19 mortalities. Moreover, according to the analysis of 3089 counties in the US, using data until 18th June 2020, long-term exposure to PM_2.5_ was associated with an increase of 11% (95% CI: 6 to 17%) in COVID-19 mortalities, attributable to a 1µg/m^3^ increase in PM_2.5_ concentration [27]. These researchers detail the role of PM as a trigger in COVID-19 spread and mortalities and describe how public policies aimed at sustainable development, such as reductions in industrial and urban emissions, had positive effects on health outcomes, reducing mortality rates and the burden on healthcare systems.

## 6. COVID-19 Transmission Dynamics

COVID-19 transmission dynamics must be scrutinized. COVID-19 transmission dynamics are essentially defined by the original reproduction count, real-time effectual reproduction count, and rates of death, which planners utilize to design measures to more successfully segregate COVID-19 carrier persons from the general population [170].

According to preliminary studies, locations with higher altitudes, colder climates, and better socioeconomic conditions, such as those in parts of North America and practically all Asian and European countries, observed more COVID-19 cases [171]. Likewise, numerous researchers have investigated the relationship between demography, environment, climate, and health risk determinants of cities/regions and COVID-19 incidence to uncover spatial-temporal variability and regulate the control of COVID-19 dissemination worldwide [28]. Environmental forces are generally categorized as natural and anthropogenic [172], and both are important for COVID-19 and SARS-CoV-2 viral transmission [173].

Several comprehensive indices, such as interactional commerce, urban sprawl, market growth, and transportation, can adequately explain COVID-19 severity [174]. The level of PM pollution changed with several lockdown-dependent parameters, as it did with COVID-19. India, for example, has experienced a significant decrease in the index of retail and leisure activities, transport hubs, and workspaces [175], resulting in a significant reduction in AQI values in Indian cities (Figure 2).

Seasonal evidence shows that meteorological variables such as temperature and surface radiation are also related to the original reproduction count of COVID-19 patients. As an airborne transmission pandemic, the severity of SARS-CoV-2 and COVID-19 infection has been found to be impacted by climate and air pollutants [176]. When viruses adhere to inanimate things, temperature and humidity affect their survival and persistence [177]. The ideal temperature and ultraviolet sun index significantly impact virus transmission and community illnesses [178]. Wind velocity, precipitation, and air pressure can all affect SARS-CoV-2 survival in the air, which may explain the high prevalence of COVID-19 in countries with stable meteorological conditions [179,180]. Previous studies revealed that climatic factors such as temperature and wind speed have a delayed effect on COVID-19 and SARS-CoV-2 patients [181]. In addition, aerosol and fomite transmission of SARS-CoV-2 is feasible [182]. Coccia proposed that “air pollution-to-human transmission,” rather than “human-to-human transmission,” is the major factor accelerating COVID-19 transmission dynamics [134].

Evidence implies that socioeconomic factors and infection management strategies impacted COVID-19 outbreaks more than meteorological variables [173]. As a result, considering health, social, and economic indices is critical to understanding lockdown-related fluctuations in the PM pollution of ambient air.

### Social Aspects

SARS-CoV-2 accesses host cells via ACE2, which is found in the human body [151]. Because SARS-CoV-2 infects people by joining ACE2, the COVID-19 infection has no regard for age, race, or gender [183]. Disparities and inequalities in health have been emphasized in the COVID-19 pandemic due to financial issues and inequalities in access to health treatments [184]. COVID-19 outbreaks are widespread in crowded settings, such as densely populated cities and transit hubs, because the disease is passed from person to person [129]. Various nations’ officials have used social lockdowns to limit COVID-19 transmission patterns, with surprising success [185,186]. According to research, the Community Mobility Index, which analyzes the behaviors of schools and colleges, travel, commerce, and social venues, fell drastically in the middle of 2020 and effectively disseminated environmental pollutants [175].

Furthermore, Figure 4A demonstrates that many metropolises that had a poor AQI–PM_2.5_ score before the pandemic were improved during the first quarter of 2020, with the enforcement of COVID-19 limits and related variables. According to studies, using a home office during the COVID-19 era might significantly reduce transport and travel, hence cleaning the atmosphere [187]. Figure 4B shows the reduction in PM_10_ pollutants. Although PM_10_ concentrations in ambient air are frequently higher than PM_2.5_, the PM_2.5_ level is more severe in metropolitan areas.

The COVID-19 outbreak has caused a global economic crisis. COVID-19 caused the most devastating global recession over the last 80 years, with a 5.2% decline in world GDP in 2020, as per Global Economic Prospects, June 2020 [188]. Furthermore, COVID-19-related segregation efforts resulted in massive economic losses [189]. For example, addressing and avoiding the COVID-19 outbreak inflicted a major financial strain on the Chinese government [190]. Because of the restrictions on people’s mobility, Italy’s lockdown policy hampered virtually all trade and commerce [174]. The COVID-19 closures reduced the reachability of trained or labor workers in New Zealand and Australia, affecting market dynamics [191].

## 7. Opportunity Cost of Lockdown

Since the SARS-CoV-19 pandemic, countries have implemented a number of non-medical interventions (NMIs) (e.g., lockdowns, stay-at-home orders, and mask mandates) to limit COVID-19 transmission. A measurable improvement in AQI in cities worldwide is a prerequisite of NMI implementation [192]. As a result, the top 50 most populated megacities in the world had an aggregate 12% improvement in atmospheric cleanliness [192], with some projections varying from 10 to 43% decreases in PM_2.5_, albeit under severe meteorological events [59]. It is projected that 3970–8900 premature causalities could be avoided each year if the ensuing cleaner air in California alone was observed last year [193]. COVID-19 prevention and mitigation measures reduced PM_2.5_ values in 20 of the 46 countries studied (PM_2.5_ concentrations were lowered by 7.4–29.1 g m^3^). COVID-19′s standard precautions, in particular, led to a significantly reduction (5.6–29.1 g m^3^) in PM_2.5_ levels across all developing countries, smaller decreases (4.6–11.3 g m^3^) in PM2.5 levels across five developed countries, and rises (1.8–7.4 g m^3^) in PM_2.5_ levels across three developed countries.

Given the health hazards posed by PM_2.5_, this improvement in the AQI will be significant for healthcare policymakers. The highest levels of AQI–PM_2.5_ were recorded in Patna, Delhi, and Dhaka in 2019, while Lucknow, Delhi, and Dhaka had the highest values in 2020. Three of the four cities mentioned are in India, with the fourth in Bangladesh, and all have very poor air quality and income. Aside from the large transportation fleet [50], domestic fuels such as wood and dry waste in these cities [194] contribute to the high PM concentration.

Furthermore, in low-income or developing countries where the economy is sluggish and pollution regulations are not adequately implemented, the AQI in cities is often detrimental [195], which is consistent with other findings of their study. There were statistically significant variations in PM_2.5_ values produced by the control procedures between developed (95% confidence interval (CI): 2.7–5.5 g m^3^) and developing nations (95% CI: 8.3–23.2 g m^3^). The COVID-19 lockdown decreased the number of fatalities and hospitalizations in the 12 developing countries by 7909 and 82,025 cases, respectively, and by 78 and 1214 cases in the eight wealthiest countries. In addition, the COVID-19 lockdown lowered the financial impact of the PM_2.5_ health burden by USD 54 million in the 12 developing countries and by USD 8.3 million in the eight advanced nations. The discrepancy was caused by variations in the chemical characteristics of PM_2.5_. Because the levels of primary PM_2.5_ (e.g., BC) in developing regions were 3 to 45 times greater than in prosperous nations’ cities during the COVID-19 lockdown, the PM_2.5_ level was more sensitive to reductions in local emissions in underdeveloped countries. On the other hand, wealthier countries have more significant mass proportions of secondary PM_2.5_ than emerging countries. As a result, these countries were more vulnerable to secondary atmospheric transmission, which may have been exacerbated by lower local pollution.

Different responses to reducing emissions imply that industrialized and developing nations should employ distinct approaches to air pollution prevention. As forecasted, the world’s 10 most polluted cities are concentrated in emerging nations [196]. Poor air quality can have detrimental consequences on human health and obliquely retard GDP recovery [197]. Shifts in AQI during the COVID-19 shutdown indicated that mitigation methods might have instant consequences on the atmosphere. Therefore, there is an immediate necessity to tackle air pollution in emerging countries.

However, most developing nations are experiencing substantial economic growth [198]. Air quality has been compromised to stimulate the domestic financial system and other priorities of state and local administration [3,199]. Noting that air quality and economic progress should not be irreconcilable is necessary. The Chinese government has made enormous attempts to curb pollution, yet this has been followed by economic growth in recent years, as indicated by the country’s gross domestic product [200]. In actuality, the economic gains of reducing air pollution might outweigh the costs [201]. Hence, wealthy nations should adopt more state-of-the-art methods of air pollution control, while emerging countries should demonstrate that economic development and air pollution control are not mutually exclusive.

## 8. Scope and Long-Term Prevalence of Lockdown

Because air pollution downturn events are uncommon (for example, the 2020 SARS-CoV-19 pandemic—“The Great Lockdown”, or the 2008 Economic Crisis—“The Great Recession”), little is recognized about how such scenarios alter the proportion of AQI in a local context or whether such adjustments have significant policy consequences [202]. Long et al. [203], for example, show that the 2008 financial crisis had a significant and negative influence on national atmospheric pollution in the United States, even after accounting for various factors. However, what is the source of this pattern? Is it true that a decrease follows every instance of pollution decline in air quality? Furthermore, the clinical characteristics of COVID-19 victims show that some classes of individuals are disproportionately clustered in terms of gender, ethnicity, age, and socioeconomic status [193]. Numerous types of research have shown that those with a background of pre-existing conditions, such as hypertension or diabetes, are at a higher risk of dying from COVID-19 [204]. Without a doubt, the preponderance of urban and industrial towns are exposed to PM pollution [205], and according to the current study’s findings, the AQI–PM_2.5_ during the lockdown phase in 2020 has substantially improved (Figure 4). These findings apply to outdoor conditions; they may vary in indoor spaces due to distinct physicochemical interactions and environmental conditions in indoor locations [206,207]. Other studies found a decline in PM_2.5_ and PM_10_ from January to the end of May due to lockdown and broad limitations in nations [82,208].

However, the COVID-19 outbreak had irreversible effects on human cultures. It was able to enhance atmospheric conditions in most areas by imposing executive restrictions in various countries. When compared to 2019, AQI readings for PM_2.5_ and PM_10_ decreased in around 83% and 86% of metropolises, respectively, in 2020. Furthermore, the data showed that AQI levels for PM_2.5_ and PM_10_ were typically higher in 2021 than in 2020, owing to a reduction in national level limits (4–7%) [104]. In general, implementing strict rules linked to COVID-19 limits can demonstrate a country’s executive capacity to reduce pollution in non-crisis scenarios. Even though this quality improvement was only temporary, it is an essential finding that health authorities can use to enhance air quality and improve human health.

## 9. Conclusions and Future Studies

A brief overview of PM air pollution, its sources, components, formation mechanism, meteorological influence on PM characteristics, and health effects concerning COVID-19 is given in this review. By evaluating all the information, PM pollution and its severity were clearer. The fine-fraction PM (PM_2.5_) is more toxic than PM_10_, as it can penetrate deeper into the lungs and cause severe health effects [131,209]. The toxicity of PM is enhanced many times due to associated chemical species [210]. The chemical characteristics of PM are directly related to emission sources, which further depend on the area’s socio-economic, weather, and geographical conditions. A further detailed assessment of various emission sources’ chemical profile, the relationship between indoor air pollution to outdoor pollution, and the evaluation of different interdisciplinary approaches for PM pollution monitoring and control may be a helpful strategy for the future. Overall, significant advantages can be achieved by greening the transport system and eliminating emissions from heavy industry, depending on background factors and sources of pollution [53]. Nevertheless, as evidence of the increased ozone concentration indicates, it is also important to consider the secondary effects of such steps. This should be discussed further in future studies.

Further studies must be conducted to better understand the role of those weather conditions that have been largely overlooked in the related literature. This is important since a modeling study found in India that while PM_2.5_ decreased during the COVID-19 lockdown, it can also increase under unfavorable weather conditions [59]. Additionally, a significant outcome would be improving air quality, which ultimately lowers transmission rates and increases citizens’ coping ability. However, this is not yet well investigated and requires further research.

The scientific evidence from previous studies highlights the substantial influence of chronic air pollution exposure on COVID-19 spread and mortality, although the possible impact of airborne virus vulnerability has not yet been modeled. PM_2.5_ and NO_2_ levels tend, in particular, to be more closely related to COVID-19 than PM_10__,_, and their association with COVID-19 mortality and incidence may be attributed to the impossibility of contacting alveolar type II cells with a PM greater than 5 μm, where the cell input receiver for SARS-CoV-2, angiotensin-converting enzyme 2 (ACE2), is located. In addition, different protocols in different countries, like different lockdown rules, infection stages, air pollution levels, topographical, socioeconomic, and sociodemographic factors, and weather, can lead to different results. While most updated studies support the correlation between air pollution and COVID-19, the limitations of this study are the limited number of publications collected and the range of methods used, which often lack findings that are difficult to compare. The first people to study this link didn’t always consider all of the confounding factors, such as control politics, rates of urbanization, availability of medical services, weather, lifestyle, population size, and socio-demographic or socio-economic variables; a global crisis forced them to work hard and analyze quickly.

Furthermore, to date, epidemic data in all countries and rates of mortality are underestimated. However, the cases included in the literature cannot be considered definitive. More research is required to improve air pollution during the COVID-19 pandemic, particularly studies evaluating the effects of multiple pollutants or multidisciplinary trials, to strengthen scientific evidence and support findings applicable to pandemic application strategies that effectively prevent new health crises. Nevertheless, reducing outdoor and indoor air pollution has provided immediate health advantages. In fact, the global health emergency demonstrates that environmental science is a fundamental metric for enhancing awareness of infectious diseases and that every intellectual and economic resource must be devoted to accelerating efforts to enforce environmental policies to reduce air pollution and implement new urban planning.

## Figures and Tables

**Table 1:** Impact of lockdown on PM pollution.

PM	Country	Location	Period	Findings	References
PM_2.5_ and PM_10_	India	Delhi and Kolkata	From 22 March to 3 May 2020	Lockdown reduced 59 and 43% PM_10_ and PM_2.5_ in Delhi and 49 and 50% in Kolkata compared to PM_10_ and PM_2.5_ concentrations found in 2019.	[81]
PM_2.5_	India	Kolkata, Mumbai, Chennai, Hyderabad, and New Delhi	From 25 March to 31st May 2020	Peak hour (i.e., 07:00–11:00 h) concentration of PM_2.5_ reduced by 63.4%, 56.4%, 48.5%, 23.8%, and 21.3% in Kolkata, Mumbai, Chennai, Hyderabad, and New Delhi by the lockdown.	[82]
PM_2.5_	India	Delhi	From 25 March to 30 April 2020	Compared to pre-lockdown, PM_2.5_ concentration decreased by 40%; 94.44% days were observed below the NAAQS 24 h standard limit of 60 μg/m^3^.	[83]
PM_2.5_	India	Bengaluru	Daily PM_2.5_ levels for 53 days. 1 March to 22 April 2020	PM_2.5_ reduced by ~15–22%.	[84]
PM_2.5_	9 most COVID-19-affected cities	New York, Los Angeles, Rome, Mumbai, Delhi, Dubai, Beijing, Shanghai, and Zaragoza	March 2020	Comparing March 2020 with March 2019, PM_2.5_ concentrations decreased in Beijing and Shanghai (up to 50%), in Delhi (35%), New York (32%), Mumbai (14%), Dubai (11%), and Los Angeles (4%). No change in Zaragoza and Rome.	[85]
PM_10_ and PM_2.5_	Malaysia and Southeast Asia	Malaysia	March-April 2020	PM_10_ and PM_2.5_ were reduced by 28–39% and 20–42% in the industrial area, and by 26–31% and 23–32% in urban areas, respectively.	[58]
PM_10_ and PM_2.5_	Southern European cities and China	Nice, Valencia, Rome, Turin, and Wuhan	1 January to 18 April 2020	PM_2.5_ and PM_10_ were reduced by ∼42% in Wuhan, by ∼8% in Europe, and ∼6% in Southern Europe.	[86]
PM_2.5_	Kazakhstan	Almaty	19 March to 14 April 2020	PM_2.5_ declined 21% with a 6–34% spatial variation.	[87]
PM_10_ and PM_2.5_	India	Delhi	1 January to 31 March 2020	PM_10_ and PM_2.5_ levels significantly reduced. Sharp decline of up to 200% of PM_2.5_ and PM_10_ concentrations.	[88]
PM_2.5_	India	Lucknow and New Delhi	1 February to 21 February and 25 March to 14 March 2020	Lockdown resulted in a significant decline in PM_2.5_.	[89]
PM_2.5_	Northern China	Beijing, Wuhan, and Northern China	23 January to 29 February 2020	PM_2.5_ decreased by 29 ± 22%. Similar reductions in PM_2.5_ (31 ± 6%) were noted in the urban area of Wuhan.	[90]
PM_10_ and PM_2.5_	China	366 Cities	24 January to 9 February 2020	A substantial decrease in PM_2.5_ and PM_10_ was attributed primarily to reduced activity in the transportation, industries, and industrial sectors. In China, PM_2.5_, decreased from 65.0 μg m^−3^ to 51.4 μg m^−3^ during lockdown. In total, 315 of the 366 cities experienced a decrease in PM_2.5_.	[91]
PM_10_ and PM_2.5_	Italy	Milan	9 March to 5 of April 2020	PM_10_ and PM_2.5_ levels were significantly reduced primarily because of reduced vehicular emissions. PM_10_ reduced up to 59% while PM_2.5_ decreased up to 47.4%.	[92]
PM_10_	Morocco	Salé City	11 March to 2 April 2020	There was an outweighing of locally emitted PM_10_ reductions by long-range transported aerosols. Overall, 75% reduction in PM_10_ concentration was reported.	[43]
PM_10_	India	Dwarka river basin within Jharkhand and West Bengal	28 March to 13 April 2020	As a result of the lockdown, PM_10_ concentrations dropped from 189–278 μg/m^3^ to 50–65 μg/m^3^.	[93]
PM_2.5_	Pakistan	Four major cities of Lahore, Islamabad, Karachi, and Peshawar.	23 March to 15 April 2020	Satellite observations reveal PM_2.5_ pollution levels reduction of 13% to 33%, whereas ground-based observations reveal 23% to 58% decrease.	[94]
PM_2.5_	Pakistan	Lahore, Karachi, Peshawar Islamabad	22 March to 30 June 2020	Pre-lockdown: 176.0, 142.5, 148.9, and 131.7; In lockdown: 108.9, 78.0, 97.2, and 83.0; Relaxed period: 133.5, 77.7, 101.7, and 82.6; In smart lockdown: 134.9, 65.3, 126.9, and 103.8	[95]

## Data Availability

Not applicable.
